# Pathogenic role of S100 proteins in psoriasis

**DOI:** 10.3389/fimmu.2023.1191645

**Published:** 2023-06-06

**Authors:** Huifang Liang, Junqin Li, Kaiming Zhang

**Affiliations:** ^1^ ShanXi Key Laboratory of Stem Cells for Immunological Dermatosis, Institute of Dermatology, Taiyuan City Center Hospital, Taiyuan, China; ^2^ State Key Breeding Laboratory of Stem Cells for Immunological Dermatosis, Institute of Dermatology, Taiyuan City Center Hospital, Taiyuan, China

**Keywords:** psoriasis, S100 proteins, interaction, signaling pathways, inflammation

## Abstract

Psoriasis is a chronic inflammatory skin disease. The histopathological features of psoriasis include excessive proliferation of keratinocytes and infiltration of immune cells. The S100 proteins are a group of EF-hand Ca^2+^-binding proteins, including S100A2, -A7, -A8/A9, -A12, -A15, which expression levels are markedly upregulated in psoriatic skin. These proteins exert numerous functions such as serving as intracellular Ca^2+^ sensors, transduction of Ca^2+^ signaling, response to extracellular stimuli, energy metabolism, and regulating cell proliferation and apoptosis. Evidence shows a crucial role of S100 proteins in the development and progress of inflammatory diseases, including psoriasis. S100 proteins can possibly be used as potential therapeutic target and diagnostic biomarkers. This review focuses on the pathogenic role of S100 proteins in psoriasis.

## Introduction

Psoriasis is a chronic, adaptive immune-mediated inflammatory skin disease with a strong genetic predisposition and autoimmune pathogenic features (1–4). Immune-mediated inflammatory dermatosis is characterized by complex multifactorial etiologies and is clinically very different from each other despite sharing a chronic inflammatory background (**Table 1**). Psoriasis presents a notable burden, with about 0.2 billion people worldwide suffering from psoriasis. The prevalence of psoriasis varies from country to country (5, 6). The pathogenesis of psoriasis is attributable to many factors, including ethnicity, genetics, gene variants, and environment (7, 8).

**Table 1:** Immune-mediated inflammatory skin diseases.

Disease	Type	Prevalence	Inducing factors	Distribution	Clinical Features
Psoriasis	Plaque, guttate, inverse, pustular, erythrodermic psoriasis	At any age, affecting 2-4% of the worldwide population	Ethnicity, genetics, environment	Scalp, elbows, knees, lumbosacral area, body folds	Scaling papules and plaques,well-circumscribed, circular, red papules or plaques with a grey or silvery-white, dry scale, relapsing
Atopic dermatitis	IgE-high, extrinsic, and IgE-normal, intrinsic subtypes	Affects approximately 5-20% of children and 1-3% of adults	Genetics, barrier dysfunction Abnormal immunity, Environment	Scalp, elbows, knees, body folds, head, face	Persistent itching, allergy, eczematous lesions, relapsing
Hidradenitis suppurativa	Stage I, stage II, stage III	Mainly in the young and middle-aged women, 1% of the general population	Tobacco smoking, obesity, psoriasis,	Axillary, inguinal, perineal, pubic anogenital regions	Deep-seated nodules, abscesses, fistulae, sinus tracts, lesion recurrence, fibrotic
Bullous diseases	Pemphigus and pemphigoid	Primarily the elderly, dramatically increasing incidence rates in recent decades	Genetics, drugs, viruses, environment	Limbs, buttocks, sacral region, neck, face, scalp	Flaccid blisters and erosions, erosions heal with crusting and scaling, pruritic eruption
vitiligo	Nonsegmental and segmental vitiligo	0.5-2% of the population worldwide	Genetic, metabolic, oxidative stress, Immune responses environment	Distal extremities, abdomen, trunk, scalp, face	Selective loss of melanocytes, nonscaly, chalky-white macules.

Psoriasis primarily comprises plaque psoriasis (the common form, approximately 90% of psoriatic cases are chronic plaque-type psoriasis), guttate psoriasis (after the streptococcal infection), inverse psoriasis (flexural psoriasis), pustular psoriasis (the rare and unstable), and erythrodermic psoriasis (systemic inflammation). Histopathologically, psoriasis is characterized by infiltration of immune cells, epidermal hyperproliferation, and abnormal keratinocyte (KC) differentiation (9). The recruitment of circulating leukocytes to the epidermis and the production of pro-inflammatory factors, such as TNF-α, IFN-γ, IL-6, IL-8, IL-23, IL-1β, and IL-17A play a crucial role in the development of psoriasis (10). In addition, psoriasis can be accompanied by comorbidities such as cardiovascular diseases, hyperlipidemia, hypertension, coronary artery disease, and diabetes (11–16).

Originally, psoriasis was thought to be due to dysregulation of keratinocyte proliferation (17), increasing evidence indicates a crucial role of the immune system in the pathogenesis of psoriasis (18). Psoriasis is caused by chronic interaction between KCs and activated immune cells (19). One of the most important features of psoriatic lesions is upregulation of expression levels of KC-originated antimicrobial peptides and proteins such as S100 protein subfamily, an important multifunctional player in inflammatory dermatoses (20, 21). The expression and secretion of S100 protein in keratinocytes, leads to the production of an array of pro-inflammatory cytokines, promoting dendritic cell maturation and CD4+ T cell proliferation (22), contributing to autoimmune system activation and psoriasis pathogenesis.

## S100 protein family

The S100 protein family is a type of Ca^2+^-binding protein composed of a multigene family of low molecular mass proteins. The S100 proteins are expressed in a cell- and tissue-specific manner and engaged in multiple functions in various cell types and tissues (23, 24). The expression levels of S100 proteins are altered in various diseases such as cardiomyopathies, neurodegenerative, inflammatory disorders, and cancers (25–29). S100 proteins act as calcium sensors that regulate the function and subcellular distribution of specific target proteins participating in a variety of signaling pathways and playing a key role in diverse cellular processes such as cell proliferation, migration, differentiation, energy metabolism, apoptosis, etc. (30). In addition, S100 proteins have an extracellular activity in response to inflammatory stimuli (30–32).

**Figure 1:**
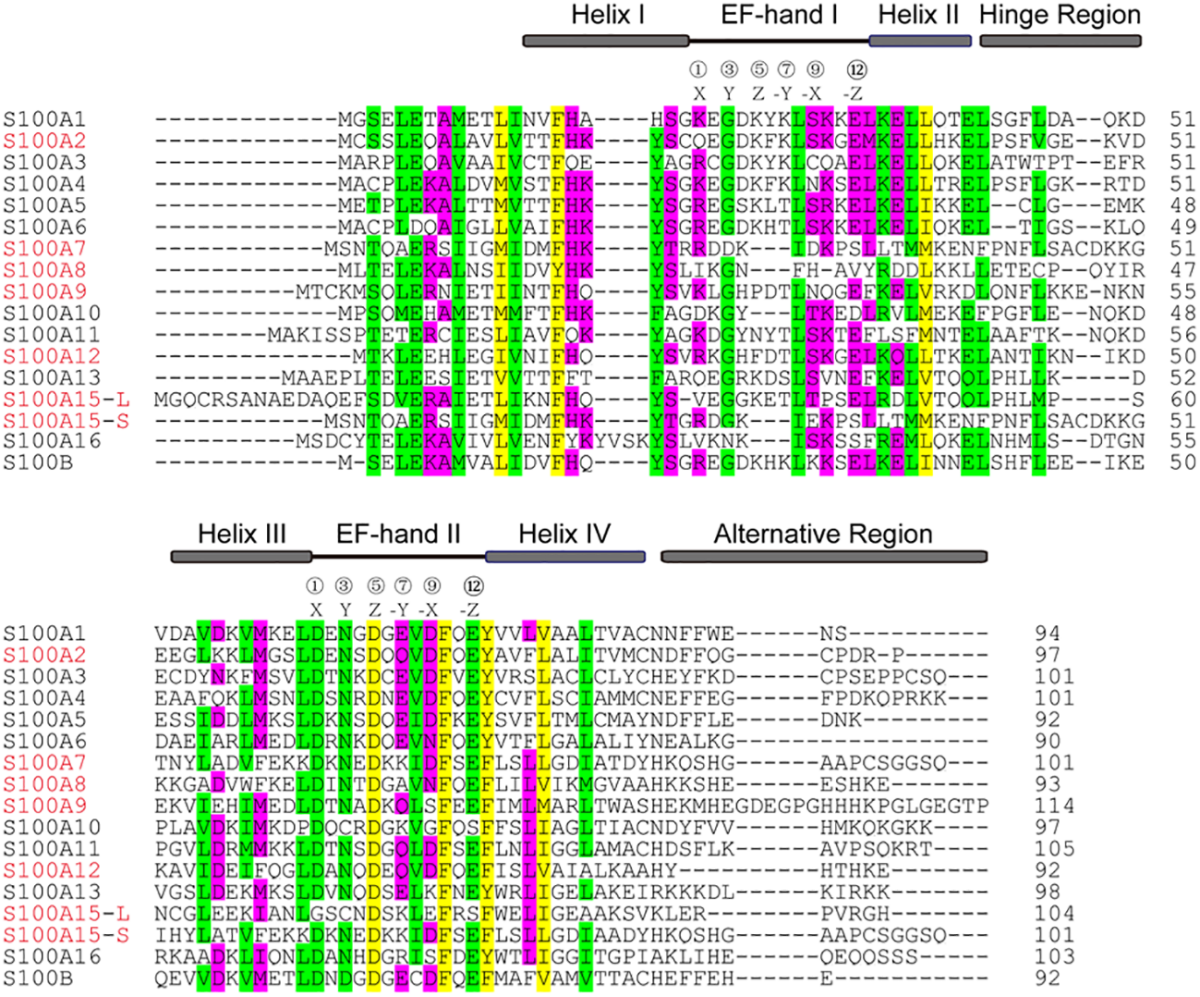
Alignment of the amino acid sequences of S100 from homo sapiens. The position of two EF-hands is indicated by black lines and the residues at positions 1, 3, 5, 7, 9, and 12 for Ca^2+^-coordination are labeled X, Y, Z, -Y, -X, -Z, respectively. Four α-helical segments, a hinge region, and an alternative region are indicated by grey boxes. The sequences highlighted in red are discussed in the text.

The S100 protein is the largest subgroup within the superfamily of calcium-binding EF-hand motif. Genes of the S100 protein family are encoded in epidermal differentiation complex (EDC) and located within the cluster on human chromosome 1q21 (33). The members of the S100 protein family have a similar low molecular weight of 10-12 kDa and share 10% - 98% similarity in the amino acid sequences. S100 proteins are typically symmetric dimers, and each subunit contains two helix-loop-helix EF-hand motifs that are separated by a flexible hinge region and flanked by conserved hydrophobic residues at the amino- and carboxy-terminal ends (34) (**Figure 1**). The calcium-binding affinity of S100 proteins is low in the absence of target proteins, but the affinity is significantly increased by several orders of magnitude in the presence of specific target proteins (35, 36). The canonical C-terminal EF-hand motif binds to calcium with 100-fold higher than the N-terminal non-canonical EF-hand (37).

The 3D structures of S100 protein family have been resolved in three different states: Ca^2+^-free apo state, bound to Ca^2+^ and bound to its target protein (38). Upon calcium binding, the S100 protein undergoes a large conformational change that creates a special target protein recognition site and allows interaction with different target proteins, including cell surface receptors and other S100 proteins (39). Moreover, S100 proteins present as a non-covalent anti-parallel homo- or heterodimers form, and the target protein can bind to opposite ends of S100 protein dimer. Thus, S100 protein dimer is a cross-bridge between two target proteins.

Because of multiple functions of the S100 proteins, many diseases, including psoriasis, are associated with altered expression levels of S100 proteins. Recent studies suggest the potential roles of S100 in keratinocyte proliferation, differentiation, and stress response. This review will focus on the expression and function of S100 protein-target protein interaction, and the signaling pathways of each S100 protein in psoriasis.

## S100A2

**Figure 2:**
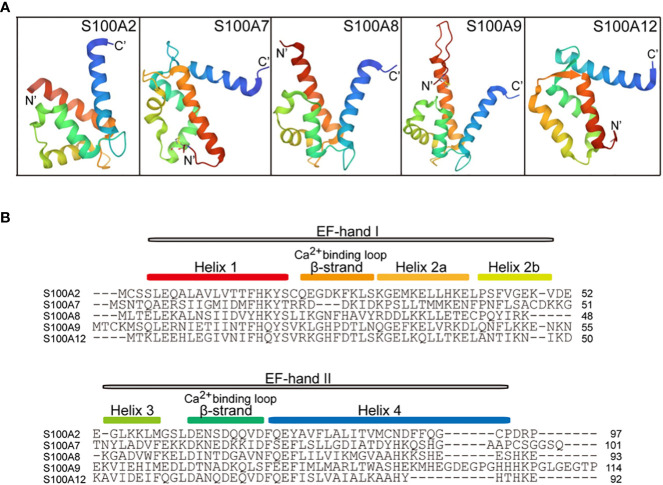
Cartoon representation of the tertiary structure in S100A proteins (PDB ID: 4DUQ, 2WOS, 1XK4, 6ZDY and 2WCF). **(A)**, comparison of the crystal structure between S100A2, S100A7, S100A8, S100A9, and S100A12. The N-terminal and the C-terminal of S100 proteins indicate N’ and C’. **(B)**, the primary secondary structure of S100A proteins. The red box indicates the helix 1 at the N-terminal, the Ca^2+^-binding loop 1, Helix 2a, Helix 2b, Helix 3, Ca^2+^-binding loop 2, and Helix 4 are indicated by the yellow box, orange box, yellow-orange box, green box, cyan box, and blue box, respectively. EF-hand regions are colored in grey boxes.

S100A2 (S100 calcium-binding protein A2) is encoded by a gene located in human chromosome 1q21 (31), which was first identified as a tumor-suppressor gene by subtractive hybridization in human mammary epithelial cells (40). The three-dimensional crystal structure of a cysteine-deficient S100A2 in the calcium-free form is similar to other S100 proteins. S100A2 contains an N-terminal specific EF-hand loop by helix I and helix II, and a C-terminal classical EF-hand motif between helix III and helix IV, which are linked by a hinge region (41) (**Figures 1**, **2A, B**). In addition to Ca^2+^, S100A2 also binds to Zn^2+^ with higher affinity, and the binding affinity of S100A2 to calcium is significantly reduced upon binding to zinc (42). Ca^2+^- and Zn^2+^-ions have opposite effects on the stability of S100A2, with Ca^2+^ acting as a protein stabilizer and Zn^2+^ as a protein destabilizer (43).

S100A2 is a member of the S100 EF-hand calcium-binding family found in mammary epithelial cells and other organs or tissues, including the lungs, kidneys, liver, and prostate gland (44). S100A2 has distinct functional roles in epithelial tissue of different origins (45). S100A2 is primarily expressed in the nucleus and moderately expressed in the cytoplasm of normal human keratinocytes (46). In the skin, S100A2 is co-localized with cytokeratin K14, an intermediate filament protein expressed primarily in basal proliferative KCs. Expression levels of S100A2 are correlated positively with the levels of cytokeratin K14. Expression levels of S100A2 gene is markedly upregulated in the involved skin of inflammatory dermatoses such as psoriasis and atopic dermatitis, and correlate positively with the severity of inflammatory skin disorders (47).

Many factors can regulate the distribution and expression levels of S100A2. For example, either oxidative stress or changes in intracellular Ca^2+^ levels enhance the translocation of S100A2 from the nucleus to the cytoplasm. Expression levels of the S100A2 gene can also be upregulated by multiple factors such as epidermal growth factor (EGF) (48), transforming growth factor β (TGF-β) (49), and interferon α (50). In keratinocyte cultures, EGF treatment significantly upregulated the expression levels of S100A2, while S100A2, but not EGF, is an effector of the regenerative hyperplasia pathway of epidermal differentiation (51). The binding of p53 to the binding site at the promoter of S100A2 can upregulate S100A2 expression (52–54). S100A2 protein is a direct transcriptional target of p73β and ΔNp63α, which both are required for the developmental and differentiation processes of keratinocytes (55). Moreover, ΔNp63α can interact with BRCA1, the breast/ovarian cancer susceptibility gene, upregulating S100A2 expression and enhancing tumor growth (56). Thus, multiple factors can regulate S100A2-mediated physiological and pathological reactions (47).

The S100A2 is an important regulator of keratinocyte differentiation, proliferation and wound healing. Epithelial-specific S100A2 transgenic mice exhibit increased proliferation and delayed skin wound repair (57). S100A2 and tumor suppressor factor p53, an important regulator in the wound healing process, can form a positive feedback loop to regulate the wound repair process. Moreover, S100A2 interacts with p53 to increase its transcriptional activity, and posttranslational modification of p53 increases its interaction with S100A2 (58). Electrophoresis and mass spectrometry assays showed a cross-link formation of S100A2 and S100A4 *via* copper-mediated oxidation of cysteine residues, resulting in increases in activation of NF-κB and secretion of TNF-α (59).

S100A2 interacts with KPNA2, a nucleocytoplasmic transport protein karyopherin α, to form a cotransport complex, transporting the tumor-associated transcription factor and regulating nucleocytoplasmic transport (60). Pulldown and coimmunoprecipitation assays revealed that the interaction between S100A2 and Hsp70/Hsp90-organizing protein or kinesin-light chain through tetratricopeptide domain modulates protein complex folding and KLC-cargo interaction (61). S100A2 can be recognized by and interact with erythropoietin, being involved in the development of tumors and other diseases (62).

**Figure 3:**
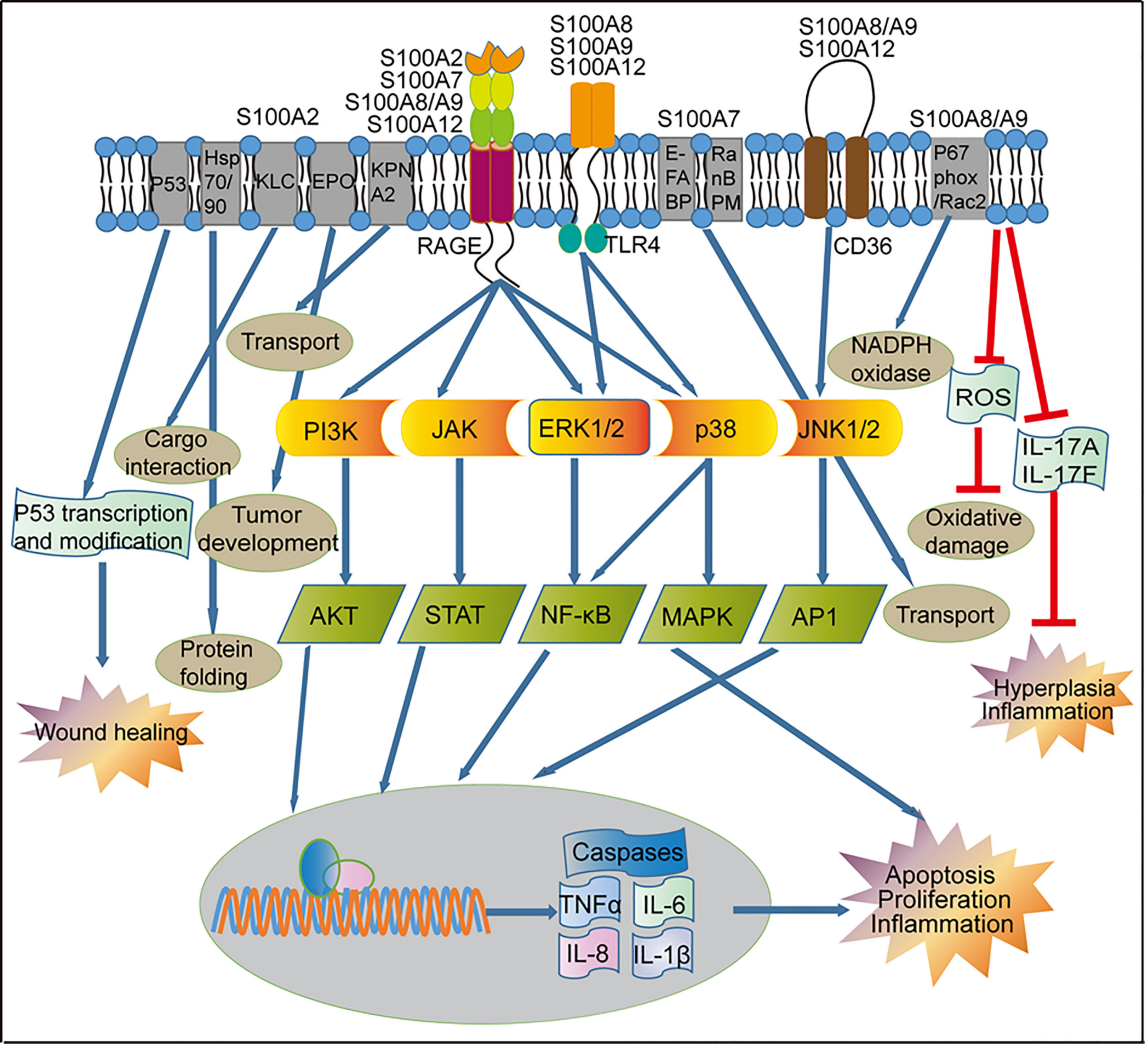
Regulation of signaling pathways by S100 proteins in psoriasis. Activation of RAGE by S100 proteins released from inflammatory cells can lead to the activation of the PI3K/AKT, JAK/STAT, and NF-κB pathways, which ultimately activate transcription of pro-inflammatory factors and other pathways that leads to proliferation and apoptosis. RAGE-mediated signaling can also activate the p38/MAPK signaling pathway in the immune cells, resulting in the up-regulated of genes involved in cell proliferation and inflammation. S100A8, S100A9 and S100A12 through the TLR4 receptor to activate NF-κB and MAPK. S100A8/A9 and S100A12 through the CD36 receptor activate the JNK1/2/AP1 signaling pathway and up-regulation of genes involved in cell inflammatory reactions. S100A2 and p53 can form a positive feedback loop to regulate the wound repair process in keratinocytes. S100A2 interacts with KPNA2 to form a cotransport complex, regulating nucleocytoplasmic transport in cancer cells, S100A2 interacts with Hsp70/Hsp90-organizing protein or kinesin-light chain, modulates protein complex folding and KLC-cargo interaction in fibroblasts. In keratinocytes, S100A7 promotes transport *via* interaction with E-FABP or RanBPM. In addition, S100A8/A9 reduces skin hyperplasia by inhibiting the production of IL-17A and IL-17F in the mouse keratinocytes. KLC, Kinesin-light chain; EPO, Erythropoietin; KPNA2, nucleocytoplasmic transport protein karyopherin α; E-FABP, epidermal fatty acid binding protein; RanBPM, Ran-binding protein M; RAGE, receptor for advanced glycation end products; TLR4, toll-like receptor 4; CD36, cluster of differentiation 36; PI3K, phosphatidylinositol 3-kinases; JAK, Janus kinase; ERK1/2, extracellular signaling-related kinase 1/2; JNK1/2, c-Jun N-terminal kinase; AKT, AGC kinase family; STAT, signal transducer and activator of transcription; NF-κB, nuclear factor kappa B, MAPK, mitogen-activated protein kinase; AP1, activator protein 1; TNF-α, tumor necrosis factor alpha; IL, interleukin.

RAGE (receptor for advanced glycation end products) is a member of an immunoglobulin protein family, including the extracellular part (composed of one variable like V-domain and two constants like C-type domains), transmembrane spanning helix, and a cytosolic domain (63). RAGE interacts with structurally diverse ligands, and plays an important role in human diseases. Although earlier studies did not show the interaction between S100A2 and RAGE, recent studies showed a micromolar affinity and strict calcium-dependent interaction between S100A2 and RAGE *via* the V-domain of RAGE (64) (**Figure 3**). However, further studies are needed to determine the functional significance of the interaction between S100A2 and RAGE.

## S100A7

S100A7, also called psoriasin, was first found as a protein overexpressed in psoriasis-involved skin and later identified as a biomarker of psoriasis-involved skin (65–67). S100A7 is an EF-hand type calcium-binding protein with a molecular weight of 11.4 kDa localized in the cytoplasm of keratinocytes and distributed at the cell periphery in terminally differentiated keratinocytes (68). The three-dimensional crystal structure of S100A7 is typically a symmetric dimer containing four α helices (69). S100A7 contains two Ca^2+^-binding domains: an N-terminal non-canonical EF-hand domain and a C-terminal canonical EF-hand domain (**Figures 1**, **2**). The amino acid and carboxyl ends are connected by a hinge region, which consists of 10-12 amino acid residues, and is crucial for target interactions. S100A7 lacks the ability of Ca^2+^-binding at the amino acid EF-hand motif, due to the absence of three amino acid residues and non-conserved substitution at position 12 of the EF-hand loop region (69). But S100A7 has a zinc-binding site composed of three histidines and an aspartate residue. The affinity of S100A7 for calcium is higher than that for zinc (70). Moreover, absence of zinc induces reorganization of the adjacent empty and distorted EF-hand loop in S100A7 structure, similar to a Ca^2+^-binding EF-hand (71). Contrary to most S100 proteins, S100A7 binding to calcium does not cause significant conformational changes.

Although S100A7 is constitutively expressed at relatively low levels in normal keratinocytes, its expression levels are dramatically increased in psoriatic lesions (72), suggesting its key role in response to inflammatory stimuli (73, 74) and the pathogenesis of psoriasis (68, 75). The altered expression of S100A7 is associated with keratinocyte differentiation and poor prognosis of psoriasis. Moreover, psoriasis-related cytokines and chemokines can upregulate S100A7 expression in normal and pathological conditions (76–81). S100A7 can also be produced by circulating cells, possibly contributing to systemic inflammation and psoriasis-associated comorbidities (10).

S100A7 exerts many functions, depending on its interaction with specific target proteins. Early studies suggested that epidermal fatty acid binding protein (E-FABP) is a candidate interaction target protein of S100A7 (82, 83). S100A7 and E-FABP form a complex participating in focal adhesion-related functions. Moreover, the S100A7/E-FABP complex binds to oleic acid to regulate oleic acid metabolism and transport. The S100A7/E-FABP complex is also involved in lipid transport and metabolism during epidermal barrier formation, and modulation of cell differentiation and migration in some dermatoses such as psoriasis (31, 68, 84).

Ran-binding protein M (RanBPM), a nucleoporin component of the nuclear pore complex, is another potential binding partner for S100A7. Both yeast two-hybrid and co-immunoprecipitation assays show an interaction of S100A7 with RanBPM, suggesting the involvement of S100A7/RanBPM complex in nucleocytoplasmic transport (85). Because expression levels of both nucleoporins Ran-binding protein 2 (RanBP2) and Ran-GTPase-activating protein 1 (RanGAP1) on the nuclear envelope are upregulated in the epidermis of psoriatic lesion, adequate expression of these nuclear envelope proteins is likely a prerequisite for nucleocytoplasmic transport in keratinocytes of the psoriatic epidermis (86).

Interaction of S100A7 with c-Jun activation domain-binding protein 1 (Jab1), a multifunctional signaling molecule, increases the activity of nuclear factor-κB (NF-κB) and phosphor-Akt (87, 88). The effects of S100A7 on NF-κB and phosphor-Akt pathway are dependent on the Jab1-binding domain and the interaction with Jab1 because mutation at the Jab1 binding domain of S100A7 does not stimulate phosphor-Akt (88). Treatment of keratinocytes with S100A7 also increases expression levels of both mRNA and protein of transglutaminase I and III *via* activation of MAPK signaling pathway. Moreover, psoriasis-involved skin displays higher expression levels of transglutaminase and alteration in the interaction between S100A7 and transglutaminase (89).

S100A7 can activate a variety of intracellular signaling pathways, and many S100A7 functions depend on the receptor for advanced glycation end products (RAGE) (90). Interaction of S100A7 with RAGE activates p38 MAPK (mitogen-activated protein kinase) and ERK (extracellular signal-regulated kinase) signaling pathways, leading to production of multiple inflammatory mediators involved in psoriasis, including IL-1α, IL-1β, IL-6, IL-8, TNF-α (91, 92) (**Figure 3**). S100A7 promotes cell proliferation and suppresses cell differentiation by inhibition of GATA3/Caspase14 (signal transducer and activator of transcription 3) signaling pathway (93, 94). Moreover, S100A7 can stimulate cell proliferation and angiogenesis through a RAGE-dependent up-regulation of endothelial growth factor (95). Recent study showed that lysine crotonylation at position 49 of S100A7 was suppressed in psoriatic lesions. Lysine crotonylation affects gene expression and epigenome, thereby affecting cell function and regulating immune responses (96, 97). Because of the important role of S100A7 in amplifying inflammatory process in psoriatic lesions, S100A7 becomes a potential diagnostic and therapeutic target for psoriasis.

## S100A8/S100A9

S100A8 (MRP8) and S100A9 (MRP14) are Ca^2+^- and Zn^2+^-binding proteins, with damage-related molecular patterns (98). Human S100A8 and S100A9 are composed of 93 and 114 amino acid residues (**Figures 1**, **2B**), respectively, and S100A9 has an isoform of 110 amino acid residues. The three-dimensional structure shows that both S100A8 and S100A9 have two helix-loop-helix motifs with charged amino acid residues (**Figures 2A, B**). The affinity of Ca^2+^-binding in the C-terminal EF-hand loop is higher than that in the N-terminal. S100A8 lacks the ability of Ca^2+^-binding in the N-terminal EF-hand motif, due to the absence of Ca^2+^-coordination necessary amino acid residues. S100A9 is different from other S100 proteins due to its long, very flexible C-terminal α-helix extension region (71).

S100A8 and S100A9 are predominantly expressed in myeloid cells (99, 100), epithelial cells (101), keratinocytes (102), and endothelial cells (103). S100A8 and S100A9 can form a stable homodimer or heterodimer by non-covalent bonding in the absence of calcium. S100A8 and S100A9 tend to form heterotetramers in the presence of calcium (104). The tetramer form is exposed to two high-affinity zinc ion binding sites (the crucial sites for functions) at the interface of the S100A8/S100A9 (calprotectin) complex (105, 106). S100A8 and S100A9 are the most abundant damage-associated molecular patterns in many inflammatory diseases. Expression levels of S100A8 and S100A9 are significantly elevated in psoriatic keratinocytes and leukocytes, and their expression levels correlate positively with the severity of psoriasis (107). The S100A8/S100A9 is a reliable biomarker for monitoring inflammatory disease activity (108–110).

S100A8/S100A9 complex plays a prominent role in regulation of inflammatory processes and immune response, including modulation of cell signaling transduction, differentiation, migration, apoptosis, regulation cytoskeleton, and so on (108, 111). S100A8/S100A9 complex promotes the polymerization of microtubules through direct interaction with tubulin, and modulates the cytoskeleton during the process of transendothelial migration (112). S100A8/S100A9 also stimulates neutrophil microbicidal activity by induction of reactive oxygen accumulation, resulting in activation of NADPH-oxidase (113). S100A8/S100A9-induced increase in NADPH-oxidase activity is *via* S100A9 transport of the cofactor arachidonic acid to the NADPH oxidase complex, and interaction of S100A8 with cytosolic *phox* proteins facilitating the enzyme assembly (114). Overexpression of S100A8/S100A9 induces cellular apoptosis (115), increases the production of pro-inflammatory cytokines and adhesion molecules, and enhances leukocyte adhesion and migration (116, 117). In addition, S100A8/S100A9 complex exerts bactericidal effects (118).

The role of S100A8 and S100A9 in the development of inflammation has been well demonstrated in murine models. For example, S100A8 deficiency induces a severe phenotype of psoriasis-like cutaneous inflammation, and rapid synchronous early resorption of the mouse embryo (119). Thus, S100A8 is an indispensable gene for mouse survival. Surprisingly, S100A9-deficient mice show slight decreases in the response of neutrophils to stimulation with chemoattractant (120) and slight aggravation of psoriasis symptoms compared with control mice (121), indicating functional differences between S100A8 and S100A9. In general, knock-out of the S100A8 or S100A9 leads to skin hyperplasia and aggravation of psoriatic symptoms in mouse models (121–123). S100A8 and S100A9 negatively regulate psoriatic skin inflammatory responses in mouse models. Combined with previous studies, it is suggested that S100A8 and S100A9 have both pro- and anti-inflammatory dualistic biological functions.

Several targets for S100A8/S100A9 have been identified, including Toll-like receptor 4 (TLR4) and myeloid differentiation factor 2 (MD 2). S100A8 interacts with TLR4-MD2 complex, enhancing inflammatory response in target cells (122) (**Figure 3**). Activation of TLR4 signaling pathway *via* S100A8/S100A9 appears to be crucial for inflammatory cascade response and systemic autoimmunity (123). In addition, S100A8/S100A9 complex interacts with p67phox and Rac2, increasing NADPH oxidase activity (114). S100A8/S100A9 complex is also involved in transcellular eicosanoid metabolism *via* interaction with scavenger receptor CD36 (124). Moreover, S100A8/S100A9 complex binds to endothelial cells *via* the S100A9 subunit interacting chiefly with heparin and heparan sulfate proteoglycans (125), contributing to the immobilization of the myeloid cell-derived S100A8/S100A9 complex on endothelium in human inflammatory diseases.

A line of evidence suggests the involvement of NF-κB signaling pathway in the action of S100A8 and S100A9. First, S100A8/S100A9 promotes tumor cell growth through the activation of the NF-κB signaling pathway in a RAGE-dependent manner (126), while S100A8/S100A9 can interact with RAGE (127). Second, amplification of pro-inflammatory cytokine response in macrophages by S100A8/S100A9 complex is *via* activation of MAPK and NF-κB signaling (128). Third, oxidative and carbonyl stresses can induce production of CML-motified (*N* ^ϵ^-carboxymethyllysine) S100A8/S100A9. The latter binds to RAGE, leading to an increase in NF-κB-dependent pro-inflammatory gene expression, suggesting that CML-S100A8/S100A9 generated in inflammatory lesions can elicit a RAGE-dependent inflammatory response (129). Additionally, carboxylated glycans promotes the binding of S100A8/S100A9 to RAGE, resulting in activation of NF-κB signaling and cell proliferation (130) (**Figure 3**).

## S100A12

S100A12, also named calgranulin C or EN-RAGE, is a pro-inflammatory protein, mainly expressed in keratinocytes of various inflammatory dermatoses (131, 132). In the psoriatic epidermis, S100A12 is expressed in the suprabasal epidermal layers (133), and its expression levels correlate positively with psoriasis severity (134). S100A12 has also been identified in neutrophils, macrophages, and lymphocytes, and functions as an innate immune defense against microorganisms. S100A12 can translocate from the cytosol to the membrane upon calcium activation.

S100A12 belongs to the S100 proteins family of EF-hand Ca^2+^-binding proteins, and its nucleotide sequence is localized within the epidermal differentiation complex on human chromosome 1q21. The predicted size of S100A12 is 92 amino acid residues with a molecular weight of 10.6 kDa (135) (**Figures 1**, **2B**). The crystal structure of S100A12 reveals that monomeric subunits have four α-helices and two EF-hand motifs linked by a hinge domain (136, 137) (**Figures 2A, B**). The N-terminal domain of the target-binding site has two residues, Glu 5 and Glu 9, that are the most highly conserved in S100 proteins (136).

The crystal structure of Zn^2+^ or Ca^2+^/Cu^2+^-bound S100A12 shows that Zn^2+^ and Cu^2+^ share the same binding site. The type of ion binding to S100A12 determines its biological function (138). Binding of S100A12 to Cu^2+^ probably is essential in early immune reactions. Intracellular S100A12 is an anti-parallel homodimer form in the presence of zinc- and calcium- ions (138–142). Extracellular S100A12 is either a homodimer or hexamer form, with cytokine-like features.

Several mechanisms are involved in the action of S100A12. Interaction of S100A2 with target proteins regulates a variety of cellular processes and is linked to certain autoimmune responses (143). S100A12 recruits both mast cells and monocytes to inflammatory sites by its flexible hinge region during early inflammatory stages (144–146). Interaction of calcium-activated S100A12 with RAGE promotes cell proliferation and pro-inflammatory responses (147–149). The binding of S100A12 to the RAGE V domain increases cellular inflammatory response to oxidative stress (150) and participates in the pathogenesis of psoriasis (**Figure 3**). Moreover, S100A12 interacts with phospholipid bilayers by specific lipid and divalent ions in physiological responses (149), suggesting that it is involved in signaling events, such as migration of monocytes, located in the cell membrane (151). S100A12 binds more closely to negatively charged lipids in the presence of Ca^2+^ or Zn^2+^, both of which can change the conformation and enhance the thermal stability of S100A12 protein, further enhancing protein-membrane interaction (152). Furthermore, interaction of S100A12 with TLR4 activates TLR4 signaling pathway (**Figure 3**), resulting in enhanced inflammatory response and migration of monocytes (153). Additionally, interaction of S100A12 with CacyBP/SIP (Calcyclin binding protein/Siah-1 interacting protein) is *via* the CD36 (a class B scavenger) receptor (154, 155). Notably, RAGE upregulates while TLR4 downregulates the expression levels of CD36 (156, 157).

S100A12 exhibits pro-inflammatory cytokine-like activities and antimicrobial activity (139, 158, 159). The antimicrobial activity of S100A12 is mainly attributed to its ability to chelate metal ions during the nutritional immunity process (160–163). The binding of S100A12 to TLR4 activates TLR4 signaling pathway, and increases the expression levels of pro-inflammatory cytokines (164, 165). Carboxylated glycans on the V-domain of RAGE enhances its binding to S100A12, subsequently activating NF-κB/ERK signaling pathway (164, 166) and upregulating pro-inflammatory molecules (165). Thus, interaction of S100A12 with its specific target proteins increases pro-inflammatory immune response and induces inflammation (143).

## S100A15

S100A15, also called koebnerisin or S100A7A, is recently identified as a member of the S100 protein family, and its amino acid sequences are 93% identical to S100A7 (167), suggesting the similarity of functions between S100A15 and S100A7. S100A15 protein is predictively composed of 101/104 amino acid residues, in which 13-48 residues contain the N-terminal non-canonical EF-hand domain, and 50-85 residues contain the canonical C-terminal EF-hand motif (168) (**Figure 1**). Interestingly, S100A15 is different from other S100 proteins because of its lack of acidic amino acids and the increased basic amino acids in the C-terminal (167).

The S100A15 gene has an alternative splicing site and two mRNA isoforms, i.e., the long isoform S100A15-L (104 amino acids) and the short isoform S100A15-S (101 amino acids) with exon 1 being spliced out (10, 90, 169). S100A15-L and S100A15-S variants are differentially expressed in normal, non-lesional and lesional skin of psoriasis. More S100A15-L than S100A15-S isoform is expressed in the skin. In comparison to normal individuals, expression levels of S100A15 are upregulated in both the uninvolved and involved skin of psoriatic subjects, with a more prominent upregulation in the involved skin.

S100A15 is an antimicrobial peptide and can increase the production and secretion of immunotropic cytokines such as TNF-α, IL-6, and IL-8 in keratinocytes, leading to the development of cutaneous inflammation (21, 170). S100A15 directly acts as a chemoattractant, promoting the infiltration of inflammatory cells and amplifying the pro-inflammatory cascade in the skin. Moreover, S100A15 act as a danger-associated molecular pattern or an alarmin factor by priming immune cells to produce pro-inflammatory mediators (168).

In contrast to S100A7, S100A15 is not able to either bind to Jab1 or interact with RAGE to activate respective signaling pathways. But it can interact with an as-yet-unknown Gi protein-coupled receptor. So far, little is known about the roles of S100A15 in multiple pathways involved in cell proliferation, migration and inflammation.

## Conclusions

The S100 protein family consists of a series of homologous proteins that are involved in a wide range of intracellular processes, including inflammatory responses. The expression levels of several S100 proteins, such as S100A2, S100A7, S100A8/S100A9, S100A12, and S100A15, are significantly up-regulated in psoriasis-involved skin. The S100 protein family interacts with specific target proteins to regulate cell proliferation, differentiation, migration, signal transduction, apoptosis, energy metastasis, and more (**Table 2**). In addition, S100 protein family plays an important pathogenic role in inflammatory diseases such as psoriasis, and can be used as a potential biomarker to estimate the prognosis and severity of diseases. However, whether the S100 protein family can be used as drug target in the management of psoriasis and possibly other inflammatory dermatoses remains to be explored.

**Table 2:** S100 proteins in psoriasis: interacting partners and associated functions.

Proteins	Target proteins	Functions
S100A2	P53	Promotes p53 transcription and post-translation modification, regulates wound repair
	KPNA2	Regulating nucleocytoplasmic transport
	Hsp70/Hsp90-organizing protein	Modulating protein folding
	Kinesin-light chain	Promoting cargo transportation
	Erythropoietin	Involved in the development of tumors
	RAGE	Not reported
S100A7	E-FABP	Participating in focal adhesion-related functions, regulating material metabolism and transport
	RanBPM	Involved in nucleocytoplasmic transport
	Jab1	Activates NF-κB and AKT, modulating progression and survival
	RAGE	Activates p38 MAPK and ERK, stimulating cell proliferation and angiogenesis
S100A8/S100A9	Tubulin	Promotes polymerization of microtubules
	P67phox/Rac2	Activates NADPH-oxidase, stimulating neutrophil microbicidal activity
	TLR4	Enhancing inflammatory cascade response in cells
	RAGE	Activates NF-κB and MAPK, promoting cell growth
S100A12	RAGE	Activates NF-κB and ERK, regulating cell proliferation and inflammatory responses
	Phospholipid bilayers	Involved in monocytes migration
	TLR4	Modulating inflammatory response and migration of monocytes
	CD36	Promotes the interaction of S100A12 with CacyBP/SIP
S100A15	Gi protein-coupled receptor	Not reported

## Author contributions

HL: data curation, visualization, writing-original draft preparation and funding acquisition; JL: reviewing and editing, funding acquisition; KZ: supervision, funding acquisition, reviewing and editing. All authors contributed to the article and approved the submitted version.
